# Tridentate *N*-Acylhydrazones as Moderate Ligands for the Potential Management of Cognitive Decline Associated With Metal-Enhanced Neuroaggregopathies

**DOI:** 10.3389/fneur.2022.828654

**Published:** 2022-02-16

**Authors:** Daphne S. Cukierman, Nicolás A. Rey

**Affiliations:** Department of Chemistry, Pontifical Catholic University of Rio de Janeiro, Rio de Janeiro, Brazil

**Keywords:** *N*-acylhydrazones, cognitive decline, biometals, metallophores, Metal-Protein Attenuating Compounds (MPACs)

## Introduction

The relevance of some endogenous metals, such as the redox-active copper and iron, and the putative neuromodulator zinc, to neurodegeneration is quite well documented in the scientific literature (1, 2). One of the pharmacological strategies that have been suggested for a chemical intervention on the bioinorganic aspects of cognitive decline involves the use of the so-called Metal-Protein Attenuating Compounds (3), or metallophores (4). These are ligands with a moderate affinity for biometals employed to prevent the oligomerization of proteins or peptides implicated in the pathophysiology of these illnesses *via* competition for binding, *in vivo*, to physiological metal ions, with redistribution and normalization of their basal levels. This approach is thus associated with metal homeostasis restoration and reduction of the oxidative stress caused by copper- or iron-containing species. On the other hand, hydrazones constitute a versatile chemical class, with a rich solution chemistry and the ability to moderately bind to transition biometals, a property that can be modulated, in terms of both affinity and specificity, by choosing the adequate substituents during the ligand planning and synthesis steps (5). Moreover, a vast range of biological activities related to this group of compounds were already described, such as antibiotic, antiviral, anticancer, vasodilator, analgesic, antidepressant, and anti-inflammatory (6–9). Interestingly, until the middle of the last decade, there were no reports about the potential use of *N*-acylhydrazones for the treatment of neurodegenerative diseases. In this opinion article, we discuss the emergence of this class as promising agents for the management (prevention and/or treatment) of cognitive decline associated with metal-enhanced aggregopathies and compare some pharmacologically important physicochemical parameters to those of other metal chelators containing the 8-hydroxyquinoline moiety proposed for the same purpose. Effectiveness of the *N*-acylhydrazone INHHQ in an animal model of Alzheimer's disease is also discussed in light of those reported for Clioquinol, PBT2 and M30 ligands.

## *N*-Acylhydrazones and Neuroaggregopathies: A Timeline

**Figure 1:**
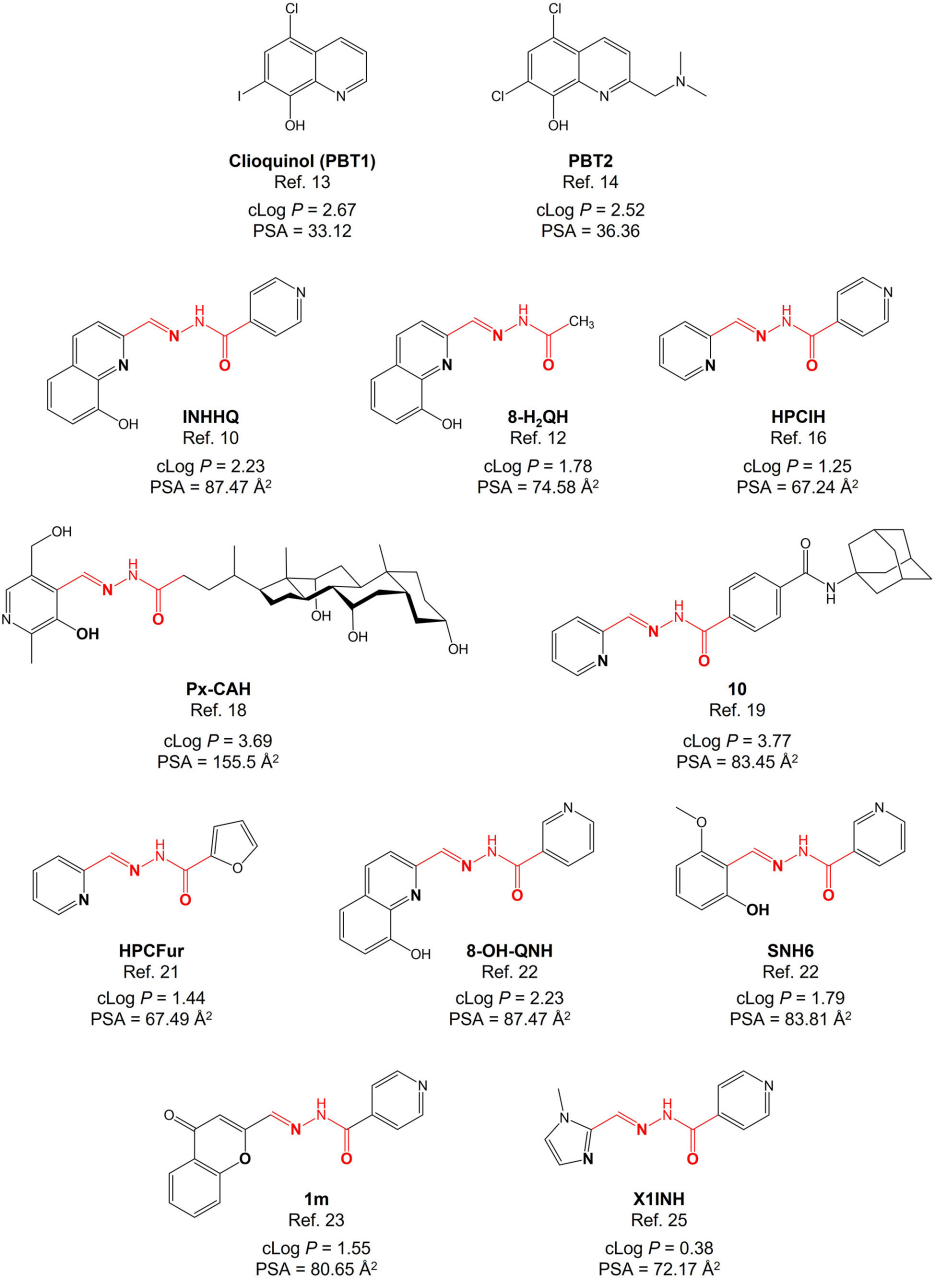
Structures of some relevant *N*-acylhydrazonic metallophores mentioned in this section, in which the characteristic functional group is highlighted in red. Clioquinol and PBT2 were included for the sake of comparison. For each compound, the calculated physicochemical parameters log *P* and PSA are also presented. Putative donor atoms of the *N*-acylhydrazones are emphasized in bold.

Figure 1 shows the structures of the main compounds mentioned in this section.

As far as we know, the first references to the potential application of *N*-acylhydrazones in the treatment of Alzheimer's disease appeared between 2013 and 2015, in works by our group (10, 11) and those of Storr and Beraldo (12). The compounds studied by these authors (INHHQ and 8-H_2_QH, respectively) still contained, in addition to the newly proposed hydrazonic moiety, an 8-hydroxyquinoline group as a legacy of pioneering metallophores Clioquinol (13) and PBT2 (14). INHHQ was shown to be able to disrupt *in vitro* Zn^2+^ and Cu^2+^ interactions with Aβ_1−40_ by a mechanism that most likely involves metal ion sequestering. The compound was not toxic to healthy Wistar rats at acute i.p. doses up to 300 mg kg^−1^ (11). On the other hand, 8-H_2_QH, which was described altogether with thiosemicarbazone and semicarbazone derivatives, is a potent antioxidant and acts inhibiting Aβ_1−40_ aggregation in the presence of Cu^2+^ ions. Native gel electrophoresis, Western blot and TEM analyses suggest that the compound also limits Aβ_1−42_ oligomerization by sequestering copper (12).

In 2017, the scope of INHHQ application was extended by us to synucleinopathies, such as Parkinson's disease and Lewy body dementia. Under this perspective, the compound presented the capacity of efficiently competing, when in an *in vitro* three-fold excess or more, with α-Syn for the binding of both reduced and oxidized forms of copper. INHHQ was non-toxic to human neuroglioma H4 cells expressing this intrinsically disordered protein and partially inhibited its intracellular oligomerization. Experiments in healthy Wistar rats demonstrated that the *N*-acylhydrazone can cross the blood-brain barrier and does not modify GSH and biometal levels in the brain, liver, kidneys and heart under a normal homeostasis condition (15). The following year, and employing the already known aroylhydrazonic iron chelator HPCIH (16) as a phenol lacking simplified chemical model for INHHQ, we concluded that the presence of the 8-hydroxyquinoline group in the ligand is not necessary for its activity as a metallophore, which seems to be only related to the *ortho*-pyridine-*N*-acylhydrazone tridentate chelating system (17). Our proof-of-concept was based on the disruption of interactions between zinc(II) ions and Aβ_1−40_ monomers.

At the time, sporadic reports by other authors on the use of this chemical class in the context of cognitive pathologies began to appear in literature. Some of them cite the works previously mentioned in here. Others look as if they were developed independently.

A publication by the Ruml and Král groups reported a new series of chelators combining metal binding and transport properties for the treatment of Alzheimer's disease. The most promising compound was Px-CAH, a tridentate *N*-acylhydrazone derived from the cholic acid hydrazide (18). On the other hand, Richarson, Kalinowski and co-workers presented novel semicarbazone and hydrazone families of adamantane-containing chelators aiming to target multiple Alzheimer's hallmarks (19). Among these, pyridine-2-carboxaldehyde (*N*-adamantan-1-yl)benzoyl-4-amidohydrazone, namely 10, was identified as a lead compound. One year later, the group of Alptuzun described a set of piperidinehydrazide-hydrazones as potential anti-Alzheimer's agents (20). Although the *N*-acylhydrazones evaluated by these authors are not tridentate ligands, so their affinity for biometals is probably low and was not verified, their acetylcholinesterase and butyrylcholinesterase inhibitory activities, as well as the ability to prevent Aβ_1−42_ self-aggregation, illustrate the versatility of the class and certainly make them worthy of reference.

A contribution from us to the Journal of Biological Inorganic Chemistry special issue “Metal Ions and Degenerative Diseases” showed that pyridine-2-carboxaldehyde-derived aroylhydrazones, as HPCFur, can play the role of peptide protecting agents toward the deleterious methionine and histidine copper-catalyzed oxidation in a mutant fragment of human PrP (21), a protein involved in a series of neurological conditions, such as the Creutzfeldt-Jakob disease. Interestingly, our experimental evidence seems to point to a mechanism different from the traditionally proposed metal ion sequestering in this case: the formation of a peptide–Cu^2+^-*N*-acylhydrazone ternary complex.

In 2020, another work from the Richardson group reported novel multifunctional iron chelators belonging to the nicotinoyl hydrazones' family for the treatment of Alzheimer's disease (22). Among them, there is an isomer of our lead compound INHHQ: 8-OH-QNH. According to the authors, the most promising substance, i.e., 6-methoxysalicylaldehyde nicotinoyl hydrazone (SNH6), markedly enhanced cellular NAD^+^/NADH ratios, promoting increased lifespan in *C. elegans*. Higher NAD^+^ concentrations are related to non-amyloidogenic APP processing (23) and protection against axonal degeneration (24) through sirtuin activation. The same year, the groups of da Silva and de Souza published data concerning the syntheses of a set of 15 isonicotinoyl hydrazones and their evaluation as potential anti-Alzheimer's drugs based on radical scavenging activities, myeloperoxidase/acetylcholinesterase inhibition and biometals chelation (25). The chromone derivative 1m was considered the best of them. By this time, we disclosed the first account about the effect of an *N*-acylhydrazone on the protection of cognitive capacities in an experimental mammal model of sporadic Alzheimer's disease. The compound INHHQ demonstrated the ability to prevent memory impairment induced by amyloid-β oligomers in mice (26).

To further improve the physicochemical characteristics of INHHQ, with the consequent enhancement in its pharmacological properties, a qualitative structure-activity study was carried out by our group. As a result of this investigation, a set of new *N*-acylhydrazones derived from 1-methyl-1H-imidazole-2-carboxaldehyde was selected due to their notable performance in terms of solubility, stability, safety and efficacy as metallophores. Among those ligands, it is worth noting X1INH (27), which presents a higher affinity for Cu^+^ than for Cu^2+^ and modulates α-Syn aggregation in a cell model of synucleinopathy. Finally, in a very recent publication, Yadav and co-workers presented a great number of carbazole-based semicarbazones and hydrazones as multifunctional anti-Alzheimer's agents (28).

## Discussion

Despite the initial progress obtained with the 8-hydroxyquinoline derivatives Clioquinol (PBT1) and PBT2 in preclinical models of Alzheimer's, bursting the scientific interest in the field, only marginal success regarding symptom improvement was observed during the clinical trials phases: overall, there was no significant difference in cognition or memory between the active treatment and placebo groups (29, 30). For this reason, most of those studies were discontinued. It is our firm opinion that the previous failures of other research efforts reflect an inappropriate chelator choice rather than a valid motive for rejecting the therapeutic metal chelation hypothesis in Alzheimer's and other forms of cognitive decline associated with neuroaggregopathies. Surprisingly, to date, many of the metallophores under investigation are still based on that archetypical pharmacophore.

Known for more than a 100 years, *N*-acylhydrazones present a bidentate chelating system constituted by the imine nitrogen and the carbonyl oxygen atoms, which can be converted into tridentate by the adequate choice of the aldehyde precursor.

*N*-acylhydrazones are well-known iron chelators and, due to this attribute, were originally proposed as agents for the treatment of iron overload diseases, including conditions such as Friedreich's ataxia, and cancer (16, 31, 32). In the context of neuroaggregopathy-related metal-triggered oxidative stress, however, copper seems to be more relevant, since *in vitro* ROS production by iron-amyloids is less efficient than that mediated by copper-amyloids. Nevertheless, *N*-acylhydrazones have proven to be efficient copper chelators as well.

Although it may seem obvious, it is important to point out that, for a drug to act in the brain, it must be able to cross the blood-brain barrier. In this sense, there are two main physicochemical parameters of interest: log *P* and PSA, the polar surface area.

Concerning the octanol-water partition coefficient, optimal values for BBB penetration are between 0 and 3 (33). On the other hand, drugs targeted to the central nervous system should exhibit a PSA of either less than 90 Å^2^ (34) or, preferentially, lower than 60–70 Å^2^ (35). Most of the *N*-acylhydrazones mentioned in the present article fit these criteria (see Figure 1). On this matter, once again, the skilled selection of the (aldehyde and hydrazide) precursors allow fine-tuning of properties, preserving the *N*-acylhydrazone functionality. For example, by including the 1-methylimidazole group in the structure of ligand X1INH, we were able to dramatically increase the water solubility of the compound conserving a more than acceptable experimental log *P*-value of 0.67 (27).

It is our belief that a lack of attention to these restrictions in the development of potential chelators for the treatment of neurodegenerative diseases might be one of the reasons behind the great number of failures observed when translating therapies from *in vitro* and *in cellulo* models to animal models and, particularly, to the human beings (clinical phase).

Regarding effectiveness in animal models, clioquinol, but especially PBT2, were found to improve learning and memory in a transgenic model of Alzheimer's disease, along with decrease in both insoluble protein content and soluble interstitial brain amyloid-β. Tests were performed with 30 or 10 mg kg^−1^ per day doses administered during 11 or 35 days, respectively (14). Another 8-hydroxyquinoline-derived chelator, the multifunctional M30 compound, is also able to attenuate anxiety and memory deficits in transgenic mice in doses of 1 or 5 mg kg^−1^ administered by gavage four times a week (36).

In contrast, with respect to the *N*-acylhydrazone INHHQ, we recently found that treatment with a single dose of only 1 mg kg^−1^ prevents both short- and long-term memory impairments in an experimental model of sporadic Alzheimer's disease (26). As far as we know, INHHQ is the first—and only—*N*-acylhydrazone evaluated regarding its efficacy in an animal model of cognitive decline so far.

Furthermore, the mechanism behind the protective activity of hydrazonic Metal-Protein Attenuating Compounds is not completely clear, and theory of simple metal abstraction is probably an oversimplification. In fact, our latest results seem to point to the formation of ternary hydrazone-metal-protein complexes as fundamental species in the prevention of protein oligomerization and redox deactivation of the metal (37). Given the facts summarized above, it is our opinion that *N*-acylhydrazones definitely deserve more attention from the researchers in the field, both for the synthesis of new derivatives with improved pharmacological profiles and, especially, for the testing of compounds belonging to this class on animal models of toxicity, efficacy and metabolism.

## Author Contributions

Both authors listed have made a substantial, direct, and intellectual contribution to the work and approved it for publication.

## Conflict of Interest

The Pontifical Catholic University of Rio de Janeiro holds granted patents in the United States of America (US 10,189,811 B2 and US 10,316,019 B2) related to the compound INHHQ, in which NR is the main inventor. Moreover, DC and NR are inventors of technology protected by the same institution through Brazilian (BR1020200054236) and international (PCT/BR2021/050107) patent applications involving 1-methylimidazole-containing *N*-acylhydrazones.

## Publisher's Note

All claims expressed in this article are solely those of the authors and do not necessarily represent those of their affiliated organizations, or those of the publisher, the editors and the reviewers. Any product that may be evaluated in this article, or claim that may be made by its manufacturer, is not guaranteed or endorsed by the publisher.
